# Optimizing thyroxine levels for enhanced buffalo sperm cryopreservation and fertility: a focus on quality, viability, and antioxidant protection

**DOI:** 10.3389/fvets.2025.1584903

**Published:** 2025-04-30

**Authors:** Maha S. Salama, Mohey A. Ashour, Ahmed M. Shehabeldin, Mohamed E. A. Omar, Mohamed M. Soliman, Heba I. Ghamry, Mohamed Abdelmegeid, Mustafa Shukry, Ahmed A. Elolimy

**Affiliations:** ^1^Animal Reproduction Research Institute (ARRI), Agricultural Research Center (ARC), Giza, Egypt; ^2^Animal Production Research Institute (APRI), Agricultural Research Center (ARC), Dokki, Egypt; ^3^Riwina Animal Production Farm, Agricultural Research Center (ARC), Ministry of Agriculture, Kafrelsheikh, Egypt; ^4^Biochemistry Department, Faculty of Veterinary Medicine, Benha University, Moshtouhor, Toukh, Egypt; ^5^Clinical Laboratory Sciences Department, Turabah University College, Taif University, Taif, Saudi Arabia; ^6^Nutrition and Food Science, Department of Biology, College of Science, King Khalid University, Abha, Saudi Arabia; ^7^Prince Sultan Bin Abdelaziz for Environmental Research and Natural Resources Sustainability Center, King Khalid University, Abha, Saudi Arabia; ^8^Department of Animal Medicine, Faculty of Veterinary Medicine, Kafrelsheikh University, Kafrelsheikh, Egypt; ^9^Veterinary Program, Faculty of Health Sciences, Higher Colleges of Technology, Sharjah Men’s Campus, Al-Ain, United Arab Emirates; ^10^Physiology Department, Faculty of Veterinary Medicine, Kafrelsheikh University, Kafrelsheikh, Egypt; ^11^Department of Integrative Agriculture, College of Agriculture and Veterinary Medicine, United Arab Emirates University, Al Ain, Abu Dhabi, United Arab Emirates

**Keywords:** thyroxine (T4), sperm cryopreservation, apoptosis-like changes, fertility rate, buffalo bull

## Abstract

**Introduction:**

This study investigated the effects of adding thyroxine (T4) to buffalo semen on sperm quality, oxidative markers, apoptosis-like changes, and fertility.

**Methods:**

Initially, we tested a wide range of T4 concentrations (0.1, 0.3, 0.9, 2.7, and 8.1 µg/dL) to evaluate their impact on motility and viability.

**Result and discussion:**

Lower concentrations (0.1–0.9 µg/dL) improved total and progressive motility and viability compared to higher concentrations (2.7 and 8.1 µg/dL). We assessed optimized doses (0.25, 0.5, 0.75, and 1 µg/dL) based on these findings. The 0.75 µg/dL group showed superior sperm velocity, viability, motion parameters, membrane, acrosome, and DNA integrity in equilibrated and frozen-thawed samples. Antioxidant markers (GPx, SOD, TAC) were enhanced, while MDA and apoptotic/necrotic cell levels were reduced, particularly in the 0.75 µg/dL group. Fertility trials revealed higher cryosurvival and conception rates in thyroxine-treated groups. In conclusion, T4 supplementation, especially at 0.75 µg/dL, enhances cryopreservation outcomes and fertility potential of buffalo bull semen.

## 1 Introduction

The thyroid gland is a crucial endocrine organ that plays a vital role in growth, reproductive activity, and metabolism by releasing thyroid hormones, including thyroxine (T4) and triiodothyronine (T3). These hormones significantly impact the male reproductive system, fertility, and sperm production (1). Sengupta and Dutta (2) demonstrated the critical role of thyroid hormones in controlling male reproductive function through various mechanisms. Additionally, T4, a metabolic hormone, has been linked to regulating energy balance and maintaining normal reproductive function in mammals (3).

Recent research has highlighted the critical role of thyroid hormones in regulating semen quality and various seminal parameters in Labrador Retriever dogs, such as semen volume, sperm motility, and viability (4). Hypothyroidism results in reduced semen volume, sperm count, poor sperm motility, and abnormal sperm morphology, while hyperthyroidism is often associated with abnormal human sperm morphology (5) and leads to a decrease in mitochondrial activity and acrosome integrity but increases plasma membrane integrity in male Wistar rats (6). In hyperthyroid rodents, the antioxidant systems are altered, with a reduction in glutathione peroxidase (GPx) and increased catalase (7). In rams, subcutaneous administration of initial doses of 300 μg _L_-T4/kg body weight per day for 3 days, followed by 25 μg T4/kg body weight per day dissolved in 1 mL alkaline saline thereafter, results in reduced testis weight and sperm motility (8).

Levothyroxine (LT4) is a synthetic form of thyroxine, primarily T4, and is the primary treatment for hypothyroidism, listed on the World Health Organisation’s essential medicines list (9). Levothyroxine supplementation before or during attempts to conceive is crucial for regulating fertility in women, reducing the risks of pregnancy loss and preterm birth in naturally conceived pregnancies (10). Several studies have introduced LT4 into semen to demonstrate its impact on sperm parameters and implications for fertility (11). Small additions of seminal LT4 (0.9 pmol L^−1^) could improve the semen sample for use in assisted reproductive techniques. In comparison, higher concentrations (9.9 pmol L^−1^) beyond physiological doses (2.9 pmol L^−1^) could harm DNA by increasing reactive oxygen species production in human sperm (12). However, LT4 reduced oxidative stress at near-physiological levels (0.9 pmol L^−1^) without affecting sperm DNA fragmentation (11).

There is currently no research examining the potential effects of different concentrations of thyroxine added to buffalo bull semen extenders on sperm functionality, quality, antioxidant capacity, and *in vivo* fertility after equilibration and post-thawing. Therefore, this study aims to investigate the impact of different thyroxine concentration additions in buffalo bull semen on the functionality, quality, and antioxidant activity of sperm after equilibration and post-thawing, as well as to improve the *in vivo* fertility of frozen–thawed semen.

## 2 Materials and methods

### 2.1 Ethical approval

The Institutional Animal Care and Use Committee of the Faculty of Veterinary Medicine at Kafrelsheikh University in Egypt approved the study, which involved sampling (IACUC#201/2021).

### 2.2 Animal management and semen collection

Seven Italian buffalo bulls, aged between 5 and 7 years old, were carefully selected for the research project. They were healthy, mature, capable of reproduction, and free from any reproductive problems or illnesses. Semen was collected twice per week using an artificial vagina (AV) maintained at 40–42°C, resulting in a total of 20 ejaculations being collected (an average of two per week). Immediately after collection, each ejaculate was assessed for volume, cleanliness, and initial motility. Only samples that met quality standards—free from contamination and with ≥70% motility were used. We did not include unclean semen samples. The buffalo bulls and buffaloes were sourced from the International Livestock Management Training Center and the Riwina Governmental Station, Animal Production Farm, and Agricultural Research Center in Kafrelsheikh, Egypt. The study occurred from March to May 2024, with the bulls being managed under regular practices during this period.

### 2.3 Assessment of thyroxine levels in the serum and seminal plasma of buffalo bulls

Blood samples of 8–10 mL were taken sterilely from the jugular vein and placed in clean glass tubes without additional substances. After feeding the animals, the samples were obtained between 8 a.m. and 10 a.m. The samples were centrifuged at 700×*g* for 15 min to isolate the serum. Afterwards, the serum samples were kept at −20°C until they were prepared for examination. After collecting semen, the seminal plasma (SP) was extracted through centrifugation at 12,000×*g* for 30 min in a cooling centrifuge at 4°C. The clear SP was then transferred into 0.6 mL microcentrifuge tubes and centrifuged again at 12,000×*g* for 10 min in a microfuge to remove any leftover sperm cell debris. It was then kept at −20°C pending thyroxine evaluation.

Thyroxine assays were conducted using specific T4 radio-immunoassay (RIA) kits (Bombay, India). The procedure, outlined with some modifications by Agarwal et al. (13), involved adding buffer, hormone-free serum, 8-Anilino-1-naphthalene-sulfonic acid (ANS), antibodies, and antigen to tubes containing unknown serum or seminal plasma samples and standards. The tubes were then incubated for 45 min at 37°C. The bound fraction was separated by adding polyethylene glycol solution (PEG) to T4, followed by centrifugation for approximately 15 min. The precipitate was used for counting. Standard curves were plotted on Logit-Log paper. The concentration in unknown samples was determined after adjusting the counts for non-specific binding using B/Bo × 100 as a response on the ordinate against log concentration on the abscissa. The assays were assessed for validity, with a 100 pg/tube sensitivity. Parallelism was tested using various dilutions of serum or seminal plasma, with a recovery rate of 115%. The intraassay coefficient of variation was 4.2%, and the coefficient of variation between assays was 10.6%.

### 2.4 Preparation of a stock solution of thyroxine

As previously mentioned by Schultze and Davis (14), with some modifications, a freshly prepared stock solution of thyroxine was created by dissolving 100 μg of levothyroxine sodium anhydrous (1 tablet of 100 μg as T4-Thyro Tablets from MUP Medical Union Pharmaceuticals, Abu-Sultan, Ismilia, Egypt) slowly and entirely in distilled water. The dissolution process was aided by adding 0.1 N NaOH, drop by drop. Once fully dissolved, the final solution was brought up to a total volume of 100 mL with distilled water, resulting in a stock concentration of 100 μg/dL of thyroxine.

### 2.5 Extender preparation and experimental design

The primary experiment was designed to evaluate a wide range of thyroxine concentrations, including both sub-physiological (0.1, 0.3, and 0.9 μg/dL) and supra-physiological levels (2.7 and 8.1 μg/dL) to assess their broad effects on sperm quality. Based on these findings, the main experiment focused on a refined range (0.25–1 μg/dL) to determine the most effective concentration within or near physiological seminal plasma levels (1.32 ± 0.09 μg/dL), thereby allowing for targeted optimization of cryopreservation and fertility outcomes.

A Tris–egg yolk-based extender was prepared using 3.028 g Tris, 1.675 g citric acid, 6.0 mL glycerol, 1.25 g fructose, and 20 mL fresh egg yolk, brought to 100 mL with double-distilled water, and supplemented with penicillin (100 IU/mL) and streptomycin (100 μg/mL), following the method described by Khalil et al. (15).

The primary experimental plan focused on varying amounts of thyroxine, with concentrations lower than seminal plasma levels (0.1, 0.3, and 0.9 μg/dL) and higher than seminal plasma levels (2.7 and 8.1 μg/dL). A three-fold serial dilution represented the control group (T0), which received no thyroxine, while the other groups received different concentrations (0.1, 0.3, 0.9, 2.7, and 8.1 μg/dL), referred to as T0.1, T0.3, T0.9, T2.7, and T8.1, respectively. This was achieved by adding 100 μL, 300 μL, 900 μL, 2.7 mL, and 8.1 mL of a stock thyroxine solution (100 μg/dL) to each 100 mL of Tris egg yolk-based extender by replacing the same amount of thyroxine with an extender to make the final volume 100 mL. Based on these findings, the main experiment optimized the most effective concentrations using a narrower range (see Supplementary Tables S1, S2).

The main experimental design involved a control extender without thyroxine (T0), while the other extenders contained varying amounts: 0.25, 0.5, 0.75, and 1 μg/dL, referred to as groups T0.25, T0.5, T0.75, and T1, respectively. This was achieved by adding 250, 500, 750, and 1000 μL of a stock thyroxine solution to each 100 mL of Tris egg yolk-based extender, replacing the same amount of thyroxine with an extender to a final volume of 100 mL.

The semen was diluted with an extender to 80 × 10^6^ spermatozoa/mL using pooled ejaculates (4–6 ejaculates) for each of the 20 runs from bulls with high sperm quality (85% normal sperm morphology and 70% motility were utilized from 95 out of a total of 140 ejaculates). For the first 35 high-quality ejaculates, the pooled ejaculate was divided into 6 portions and supplemented with varying concentrations of thyroxine extender (0, 0.1, 0.3, 0.9, 2.7, and 8.1 μg/dL) for the primary experimental design. For the second 60 high-quality ejaculates in the main experimental design, the pooled ejaculate was divided into five equal portions and supplemented with different concentrations of thyroxine extender (0, 0.25, 0.5, 0.75, and 1 μg/dL).

After dilution, the samples were chilled at 4°C for 4 h for stabilization. Subsequently, the samples were loaded into 0.25 mL polyvinyl chloride straws (IMV, L’Aigle, France). The straws were then placed in a biofreezer (Mini Digit-cool, ZH 400, IMV Technologies, L’Aigle, France) and frozen at −140°C. This was achieved by applying a freezing rate of −30°C/min (from 4°C to −15°C) and −50°C/min (from −15°C to −140°C) before being immediately submerged in liquid nitrogen for storage (16). The cryopreserved straws were thawed at 37°C for 30 s in a water bath for evaluation.

### 2.6 Equilibrated and post-thawed semen evaluation

#### 2.6.1 Sperm motility, kinematics, viability, and abnormalities

A computer-assisted system, the Computer-Aided Sperm Motion Analyzer System (CASA) from Hamilton-Thorne Biosciences in Beverly, MA, United States, was used to measure sperm motility and kinematics precisely. The system categorized sperm speed into four groups: fast (> 80 μm/s), medium (> 60 μm/s), slow (> 20 μm/s), and static. Various parameters were evaluated, including total and progressive motility percentages, straight linear velocity (VSL), average path velocity (VAP), curvilinear velocity (VCL), linearity (LIN), straightness (STR), and beat cross frequency (BCF). Wobble coefficient (WOB) was calculated based on the collected data (17). Additionally, five microliters of semen were gently added to a clean and pre-warmed Makler chamber at 37°C, with eight random microscopic fields being analyzed during each assessment (see Supplementary video).

Semen smears were prepared by placing drop (5 μL) of semen onto a microscope slide pre-warmed to 40°C and mixing it with an equal volume of staining solution. The staining solution consisted of one part 5% bluish eosin (Carl Roth GmbH + Co. KG, Karlsruhe, Germany) and four parts 10% nigrosin aqueous solution (Sigma-Aldrich, Saint Louis, MO, United States). A glass rod was used to gently spread the mixture into a uniform smear. Slides were then air-dried a Bunsen flame. An Olympus Bx-53 light microscope from Tokyo, Japan, was used at 400× magnification to distinguish between live and dead sperm cells following (18–20). The same slide was used to evaluate sperm abnormalities, such as the double tail, broken tail, coiled tail, terminally coiled tail, pear-shaped head, microcephalic head, round short head, double head, and loosehead, using an oil immersion lens (21). At least 200 spermatozoa were counted across five microscopic fields to assess abnormalities and viability.

#### 2.6.2 Plasma membrane integrity

To assess the functionality of the sperm plasma membrane, we conducted the hypo-osmotic swelling test (HOST), as described in a study by Kumar et al. (22). The test involved combining 10 μL of semen with 100 μL of a hypo-osmotic solution with a concentration of 150 mOsm/kg (0.0735 g of sodium citrate and 0.1351 g of fructose dissolved in 10 mL of Milli-Q water). The mixture was then placed in a water bath at 37°C for 30 to 60 min. Following incubation, the sample was examined under a phase-contrast microscope at 400× magnification on a heated slide (38°C) with a coverslip to observe any coiling or bending of sperm tails. Sperm cells exhibiting coiled or expanded tails were deemed to have intact plasma membranes (HOST-positive).

#### 2.6.3 Assessment of acrosome integrity

Acrosome integrity was assessed using a modified Giemsa staining technique, incorporating trypan blue pre-treatment as described by Santos et al. (23). A 200 μL aliquot of diluted and frozen–thawed semen was mixed with 0.2% trypan blue in a plastic tube and incubated in a water bath at 37°C for 10 min. The sperm were then treated with 2 mL of modified Brackett and Oliphant medium without albumin and centrifuged at 700×*g* for 6 min (24). After discarding the supernatant, the spermatozoa pellet was resuspended in the same medium, and the process was repeated until the suspension turned either light blue or clear. A small sperm suspension (10–20 μL) was placed on a glass slide and spread thinly using another slide. The slides were then dried on a heating stage at 40°C and stained with a 10% Giemsa solution (freshly prepared) for 1 h. Afterward, the slides were rinsed with distilled water and left to dry. Approximately 100 sperm cells were randomly selected per slide and examined under bright-field microscopy. Intact acrosomes appeared pink or purple, while damaged acrosomes appeared white or grey.

#### 2.6.4 Assessment of mitochondrial activity

The mitochondrial function of sperm cells was evaluated using the JC-10 Assay for Flow Cytometry (25), a lipophilic cation provided by Sigma-Aldrich (USA). The JC-10 dye changes color from green to orange in response to variations in mitochondrial membrane potential (MMP). Sperm samples, whether equilibrated or thawed, were prepared by placing 1 × 10^6^ sperm/mL into polypropylene tubes. These samples were then treated with JC-10 (500 μL) and incubated at 37°C for an hour before being washed with PBS (1 mL) and centrifuged (10 min at 800×*g*). The resulting pellet was resuspended in PBS (1 mL) and subjected to flow cytometry analysis. Emission filters of 595 and 535 nm were used to quantify the orange and green fluorescence of the sperm populations. Green fluorescence signified a lower mitochondrial membrane potential, while orange fluorescence indicated a higher potential (25).

#### 2.6.5 DNA fragmentation

DNA integrity was examined using the acridine orange assay, following the method outlined by Martins et al. (26) and Salama et al. (25). Semen samples were spread on glass slides and preserved overnight in Carnoy’s solution (a mixture of methanol and glacial acetic acid in a 3:1 ratio). The slides were dried and soaked in a tampon solution (15 mmol/L Na_2_HPO_4_, pH 2.5, and 80 mmol/L citric acid) for 5 min at 75°C. Subsequently, the slides were stained with a 0.2 mg/mL acridine orange solution. After rinsing the slides with water, they were covered and examined under an epifluorescence microscope. One hundred cells were analyzed from each semen sample. Cells with normal DNA content emitted a green fluorescence, while those with abnormal DNA exhibited fluorescence in various shades, ranging from yellow-green to red (25).

#### 2.6.6 Cryosurvival rate (%)

The cryosurvival rate of frozen–thawed buffalo spermatozoa was determined by calculating the percentage of post-thaw total motile sperm compared to pre-freeze total motile sperm, as reported in a study by Nagata et al. (27).

#### 2.6.7 Apoptosis-like changes

The Annexin-V and PI kits from IQP in Groningen, Netherlands, were used to analyze apoptotic changes in sperm by detecting phosphatidylserine (PS) externalization following the manufacturer’s instructions. The sperm samples were washed with calcium buffer, then 10 μL of Annexin-V FITC was added, and the samples were incubated on ice for 20 min. Next, 10 μL of propidium iodide (PI) was added to the sperm suspension and incubated on ice for 10 min. The sample was evaluated using flow cytometry with an AccuriC6 Cytometer (BD Biosciences, San Jose, CA, United States) and software from Becton Dickinson.

Using flow cytometric methods, the sperm subpopulation was classified into four distinct groups: Group I contained viable, non-apoptotic cells that tested negative for both Annexin-V and PI; Group II consisted of early apoptotic cells positive for Annexin-V but negative for PI; Group III included late apoptotic cells positive for both Annexin-V and PI; and Group IV comprised necrotic cells that were negative for Annexin-V but positive for PI (28).

#### 2.6.8 Antioxidant activity and lipid peroxidation

Frozen–thawed semen (three straws/group) was centrifuged at 1,000×*g* for 10 min. The resulting supernatants were used in quadruplicate to measure superoxide dismutase (SOD) activity, GPx activity, total antioxidant capacity (TAC), and malondialdehyde (MDA) levels following the manufacturer’s instructions provided by Cayman Chemicals Company.

##### 2.6.8.1 Total antioxidant capacity

The total antioxidant capacity was determined using an assay kit from Michigan, USA, as reported by Lone et al. (29). Initially, 10 μL of sample or standard was placed in duplicate, along with 150 μL of chromogen and 10 μL of metmyoglobin in each well of the plate. The reaction was initiated by adding 40 μL of H_2_O_2_ to the plate. The plate was then sealed and incubated at room temperature for 5 min. The absorbance at 750 nm was measured using a plate reader, and the TAC (Mm) was calculated using the equation of the standard curve resulting from linear regression (29).

##### 2.6.8.2 Glutathione peroxidase activity

The glutathione peroxidase activity was assessed using the GPx assay kit (22). In brief, each well of the plate contained 25 μL of the co-substrate mixture, 50 μL of assay buffer, and 10 μL of standards or samples, along with 10 μL of cumene hydroperoxide to initiate the reaction. The absorbance at 340 nm was monitored every minute for at least 5 min using a plate reader. Each sample’s GPx activity (nmol/min/mL) was calculated after generating the GPx standard curve.

##### 2.6.8.3 Superoxide dismutase activity

The activity of superoxide dismutase was assessed using the SOD assay kit (22). Briefly, in each well of the plate, a mixture of 5 μL of standards or samples, 100 μL of the diluted radical detector, and 10 μL of diluted xanthine oxidase was added. The plate was then gently agitated and placed at room temperature for 20 min. The SOD activity (U/mL) of each sample was calculated after a SOD standard curve was generated.

##### 2.6.8.4 Malondialdehyde content

The level of malondialdehyde was determined using the TBARS assay kit (22). In brief, 100 μL of color reagent, 25 μL of samples or standards, and 25 μL of a sodium dodecyl sulfate solution were combined in a clean test tube. These test tubes were cooled in ice for 10 min and placed in a hot water bath for 60 min. Subsequently, the mixture was centrifuged at 1,600×*g* at 4°C for 10 min. After centrifugation, 40 μL of the mixture was transferred to a colorimetric plate, and the absorbance was measured at 535 nm. The MDA concentration (μM/mL) of the samples was calculated by generating the MDA standard curve.

### 2.7 *In vivo* fertilization test

This study divided 125 healthy cyclic buffaloes into five groups, each containing 25 buffaloes. They were administered GPG synchronization with two injections of 20 μg Buserelin acetate (Receptal, Intervet International, Netherlands) at nine-day intervals, along with a single injection of 750 μg Cloprostenol sodium (Estrumate from Coopers Animal Health L.T.D., Berkhamsted, England) on Day 7.

Artificial insemination was performed for all buffaloes with duplicate inseminations (in the morning and evening) on Day 10 using 250 frozen–thawed straws containing 20 × 10^6^ spermatozoa from five different thyroxine extenders (T0, T0.25, T0.5, T0.75, and T1). The insemination procedures took place in May 2024. On Day 45 after insemination, pregnancy was confirmed in all inseminated buffaloes using linear probe ultrasonography [a real-time B-mode veterinary ultrasound device (Esaote Pie Medical Aquila Pro-Vet with 6.0/8.0 MHz LA Rectal Veterinary, Esaote, Italy)].

### 2.8 Statistical analysis

Statistical analysis of the data was conducted using analysis of variance with (30) General Linear Model Procedure. Variations in means were examined using Duncan’s multiple-range test (31). The Chi-square test (*χ*^2^) was used to analyze the pregnancy rate. All differences were deemed significant at *p* < 0.05.

## 3 Results

### 3.1 Thyroxine concentration in the serum and seminal plasma of buffalo bulls

**Figure 1:**
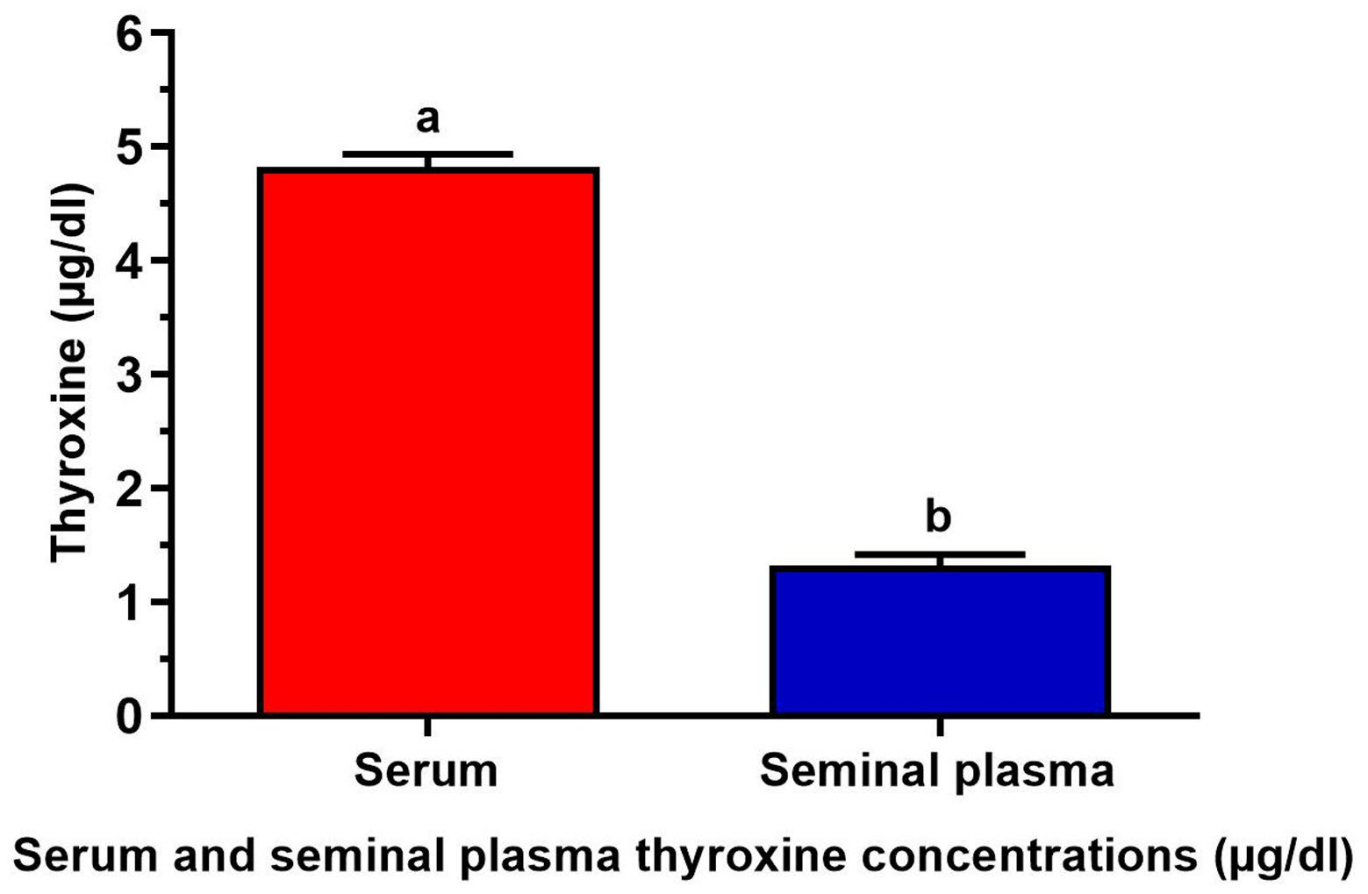
Serum and seminal plasma thyroxine concentrations (μg/dL) of 7 buffalo bulls. The data is presented as Mean ± SEM. A variety of superscripts (a, b) denote statistically significant variations within the columns where *p*-values are less than 0.05.

In Figure 1, the serum thyroxine level was measured at 4.82 ± 0.11 μg/dL, while the seminal plasma thyroxine level was recorded at 1.32 ± 0.09 μg/dL.

### 3.2 Primary experiment

#### 3.2.1 Effects of thyroxine on the viability, total, and progressive motility of equilibrated and frozen–thawed sperm

As shown in Table 1, the total motility, progressive motility, and viability of the first three lower thyroxine concentrations (T0.1, T0.3, and T0.9) were significantly higher than the last two higher thyroxine concentrations (T2.7 and T8.1) for equilibrated and frozen–thawed buffalo bull sperm.

**Table 1:** Effect of the addition of different experimental thyroxine doses on motility and viability (%) of equilibrated and frozen–thawed buffalo bull semen.

Semen	Parameters/groups	Control	T0.1	T0.3	T0.9	T2.7	T8.1
Equilibrated	Total motility (%)	75.33 ± 0.83^a^	70.89 ± 0.63^b^	75.67 ± 0.65^a^	70.56 ± 0.87^b^	55.78 ± 1.22^c^	60.44 ± 0.56^d^
	Progressive motility (%)	71.11 ± 0.68^a^	66.78 ± 1.99^b^	71.00 ± 0.91^a^	65.33 ± 1.09^b^	60.89 ± 0.63^c^	55.44 ± 0.67^d^
	Viability (%)	77.33 ± 0.97^a^	72.56 ± 0.94^b^	77.22 ± 0.74^a^	73.89 ± 0.82^b^	68.67 ± 0.69^c^	63.44 ± 0.80^d^
Frozen–thawed	Total motility (%)	45.22 ± 0.79^b^	45.89 ± 0.66^b^	55.67 ± 0.75^a^	57.00 ± 0.69^a^	40.44 ± 0.60^c^	30.78 ± 0.77^d^
	Progressive motility (%)	39.44 ± 0.63^b^	40.22 ± 0.81^b^	51.00 ± 0.87^a^	51.56 ± 0.73^a^	36.67 ± 0.87^c^	25.11 ± 0.87^d^
	Viability (%)	47.67 ± 0.83^b^	48.22 ± 0.91^b^	57.56 ± 0.80^a^	59.44 ± 1.03^a^	42.11 ± 1.07^c^	33.00 ± 0.91^d^

Number of ejaculates = 35. The means bearing one common superscript within the same row were similar (*p* ≥ 0.05).

The total progressive motility and viability of equilibrated semen were significantly higher (*p* < 0.05) in the control and T0.3 groups compared to the other groups and decreased significantly (*p* < 0.05) with higher thyroxine concentrations. There was no significant difference between the control and T0.1 groups in frozen–thawed samples. The highest values (*p* < 0.05) were found in the T0.3 and T0.9 groups, while the lowest values were significantly decreased (*p* < 0.05) with higher thyroxine concentrations in the T2.7 and T8.1 groups.

### 3.3 Main experiment

#### 3.3.1 Effects of thyroxine on motility, kinematics, viability, and abnormality of equilibrated sperm

The total and progressive movement levels in the control, T0.5, and T0.75 groups were significantly higher (*p* < 0.05) than in the T0.25 and T1 groups. Additionally, the velocity values (VAP, VCL, and VSL μm/s) were significantly greater (*p* < 0.05) in the T0.75 group compared to the other groups. However, there were no notable differences in the control STR and LIN (%) compared to the other groups (Table 2). In contrast to the different groups, the WOB (%) of the T0.5 group showed the least significance (*p* < 0.05), while the T0.75 group demonstrated the highest BCF (Hz) and viability values (*p* < 0.05). All groups had no significant variations in abnormalities (%) (Table 2).

**Table 2:** Effect of different thyroxine additives on motility (%), kinematic, viability, and abnormality parameters of equilibrated buffalo bull sperm.

Parameters/groups	Control	T0.25	T0.5	T0.75	T1
Total motility (%)	75.25 ± 0.79^a^	70.63 ± 0.80^b^	75.38 ± 0.75^a^	75.50 ± 0.90^a^	70.88 ± 0.91^b^
Progressive motility (%)	69.50 ± 0.87^a^	64.25 ± 0.86^b^	69.25 ± 0.86^a^	70.00 ± 0.82^a^	65.75 ± 0.68^b^
VAP (μm/s)	72.88 ± 0.89^b^	72.00 ± 0.78^b^	75.75 ± 0.75^a^	77.38 ± 0.99^a^	72.50 ± 0.73^b^
VCL (μm/s)	80.88 ± 0.90^bc^	80.13 ± 0.98^bc^	82.50 ± 0.82^b^	85.25 ± 0.75^a^	79.63 ± 0.82^c^
VSL (μm/s)	26.38 ± 0.62^bc^	25.75 ± 0.65^c^	27.87 ± 0.72^ab^	29.75 ± 0.68^a^	25.38 ± 0.63^c^
STR (%)	36.19 ± 0.70^ab^	35.75 ± 0.75^b^	38.43 ± 0.49^a^	36.86 ± 1.20^ab^	35.01 ± 0.84^b^
LIN (%)	32.61 ± 0.66^ab^	32.15 ± 0.77^b^	34.92 ± 0.88^a^	33.84 ± 1.08^ab^	31.90 ± 0.89^b^
WOB (%)	90.21 ± 1.68^a^	89.95 ± 1.42^a^	81.26 ± 1.11^b^	91.83 ± 0.47^a^	91.15 ± 1.57^a^
BCF (Hz)	45.13 ± 0.95^b^	44.63 ± 0.92^b^	45.25 ± 0.67^b^	50.88 ± 0.83^a^	47.00 ± 0.94^b^
Viability (%)	76.50 ± 0.71^bc^	73.13 ± 0.83^d^	76.75 ± 0.70^b^	79.25 ± 0.92^a^	73.88 ± 0.93^cd^
Abnormality (%)	9.75 ± 0.36^a^	10.13 ± 0.40^a^	10.00 ± 0.38^a^	9.63 ± 0.49^a^	10.25 ± 0.37^a^

Number of ejaculates = 60. The means bearing one common superscript within the same row were similar (*p* ≥ 0.05).

VAP, average pathway velocity (μms); VCL, curvilinear velocity (μms); VSL, straight-line velocity (μms); STR, straightness (VSL/VAP; %); LIN, linearity (VSL/VCL; %); WOB, wobble (VAP/VCL; %); BCF, beat cross frequency (Hz).

#### 3.3.2 Effects of thyroxine on the motility, kinematics, viability, and abnormality of frozen–thawed sperm

The findings displayed in Table 3 indicate a significant increase (*p* < 0.05) in total and progressive motility, as well as speed values (VAP, VCL, and VSL μm/s) in the T0.75 group compared to the other groups. Additionally, the STR, LIN, and WOB (%), along with BCF (Hz) values in the T0.5, T0.75, and T1 groups, were significantly higher (*p* < 0.05) compared to the control and T0.25 groups. Regarding viability (%), the T0.75 group displayed the highest percentage while having the lowest abnormality (%) among the other groups, as shown in Table 3.

**Table 3:** Effect of different thyroxine additives on motility (%), kinematic, viability, and abnormality parameters of post-thawed buffalo bull sperm.

Parameters/groups	Control	T0.25	T0.5	T0.75	T1
Total motility (%)	44.38 ± 1.08^c^	45.63 ± 1.09^c^	55.75 ± 0.68^b^	60.88 ± 0.90^a^	57.50 ± 0.96^b^
Progressive motility (%)	38.13 ± 0.81^c^	38.87 ± 0.79^c^	48.50 ± 0.78^b^	54.50 ± 0.89^a^	50.38 ± 0.77^b^
VAP (μm/s)	58.25 ± 0.92^c^	58.87 ± 0.85^c^	62.13 ± 0.67^b^	68.38 ± 0.71^a^	63.50 ± 0.78^b^
VCL (μm/s)	73.75 ± 0.70^c^	74.63 ± 0.82^c^	82.50 ± 0.63^b^	88.75 ± 0.70^a^	84.13 ± 0.81^b^
VSL (μm/s)	17.88 ± 0.58^c^	18.13 ± 0.58^c^	22.63 ± 0.60^b^	25.13 ± 0.55^a^	23.00 ± 0.59^b^
STR (%)	30.75 ± 1.13^b^	30.82 ± 1.05^b^	36.44 ± 1.01^a^	36.80 ± 1.05^a^	36.27 ± 1.10^a^
LIN (%)	24.24 ± 0.79^b^	24.28 ± 0.70^b^	27.41 ± 0.63^a^	28.33 ± 0.69^a^	27.38 ± 0.86^a^
WOB (%)	78.96 ± 0.69^a^	78.96 ± 1.45^a^	75.35 ± 1.15^b^	77.05 ± 0.67^ab^	75.54 ± 1.23^b^
BCF (Hz)	27.12 ± 0.69^b^	27.75 ± 0.70^b^	30.00 ± 0.68^a^	32.13 ± 0.61^a^	30.88 ± 0.90^a^
Viability (%)	46.75 ± 0.80^c^	47.88 ± 0.79^c^	57.38 ± 0.73^b^	62.00 ± 0.71^a^	58.88 ± 0.67^b^
Abnormality (%)	14.25 ± 0.45^a^	14.13 ± 0.44^a^	12.75 ± 0.37^b^	10.88 ± 0.44^c^	12.25 ± 0.37^b^

Number of ejaculates = 60. The means bearing one common superscript within the same row were similar (*p* ≥ 0.05).

VAP, average pathway velocity (μms); VCL, curvilinear velocity (μms); VSL, straight-line velocity (μms); STR, straightness (VSL/VAP; %); LIN, linearity (VSL/VCL; %); WOB, wobble (VAP/VCL; %); BCF, beat cross frequency (Hz).

#### 3.3.3 Effects of thyroxine on the functional parameters of equilibrated and frozen–thawed sperm

According to the findings in Table 4, the equilibrated semen group treated with T0.75 showed significantly higher (*p* < 0.05) levels of acrosome (%), plasma membrane (%), MMP (%), and DNA integrity (%) compared to the other groups (*p* < 0.05). In frozen–thawed semen samples, there were no significant differences between the control group and the T0.25 group. A similar trend was observed between the T0.5 and T1 groups in terms of acrosome (%), plasma membrane (%), MMP (%), and DNA integrity (%), with the highest values seen in the T0.75 group (Table 4).

**Table 4:** Effect of different thyroxine additives on function parameters of equilibrated and frozen–thawed buffalo bull sperm.

Semen	Parameters / groups	Control	T0.25	T0.5	T0.75	T1
Equilibrated	Acrosome integrity (%)	70.75 ± 0.65^c^	71.12 ± 0.55^c^	73.38 ± 0.42^b^	75.63 ± 0.49^a^	74.25 ± 0.59^ab^
	Membrane integrity (%)	57.13 ± 0.69^c^	60.38 ± 0.50^b^	60.75 ± 0.49^b^	65.87 ± 0.58^a^	61.62 ± 0.63^b^
	MMP (%)	50.25 ± 0.59^d^	51.75 ± 0.45^c^	54.87 ± 0.52^b^	57.62 ± 0.38^a^	53.13 ± 0.51^c^
	DNA integrity (%)	89.63 ± 0.65^c^	90.50 ± 0.73^c^	91.13 ± 0.44^bc^	94.63 ± 0.38^a^	92.63 ± 0.60^b^
Frozen–thawed	Acrosome integrity (%)	63.13 ± 0.58^c^	63.63 ± 0.46^c^	66.13 ± 0.48^b^	69.38 ± 0.60^a^	66.88 ± 0.39^b^
	Membrane integrity (%)	43.63 ± 0.53^c^	45.00 ± 0.66^c^	49.12 ± 0.39^b^	52.50 ± 0.57^a^	49.75 ± 0.62^b^
	MMP (%)	45.63 ± 0.49^c^	46.50 ± 0.59^c^	49.50 ± 0.42^b^	54.88 ± 0.52^a^	50.13 ± 0.55^b^
	DNA integrity (%)	86.37 ± 0.46^d^	87.75 ± 0.45^cd^	89.13 ± 0.64^bc^	91.63 ± 0.49^a^	89.75 ± 0.59^b^

Number of ejaculates = 60. The means bearing one common superscript within the same row were similar (*p* ≥ 0.05).

MMP, mitochondrial membrane potential.

#### 3.3.4 Effects of thyroxine on antioxidant parameters of post-thawed sperm

All groups treated with thyroxine, especially the T0.75 group, showed improvements in antioxidant parameters such as GPx, SOD, and TAC values compared to the control group. The only exception was the T0.25 group, which showed no significant differences in SOD levels. Regarding MDA levels, all thyroxine groups, particularly the T0.75 group, had lower values than the control group (Table 5).

**Table 5:** Effect of different thyroxine additives on antioxidant parameters of post-thawed buffalo bull sperm.

Parameters / groups	Control	T0.25	T0.5	T0.75	T1
GPx (nmol/min/mL)	15.13 ± 0.39^d^	16.63 ± 0.38^c^	20.25 ± 0.31^b^	22.88 ± 0.40^a^	20.75 ± 0.37^b^
SOD (U/mL)	32.25 ± 0.53^d^	33.12 ± 0.44^d^	35.75 ± 0.37^c^	42.38 ± 0.50^a^	37.63 ± 0.49^b^
TAC (Mm)	1.38 ± 0.03^e^	1.69 ± 0.05^d^	1.92 ± 0.03^c^	2.33 ± 0.06^a^	2.12 ± 0.03^b^
MDA (μM/mL)	1.33 ± 0.03^a^	0.99 ± 0.03^b^	0.81 ± 0.02^c^	0.49 ± 0.02^e^	0.74 ± 0.02^d^

Number of ejaculates = 60. The means bearing one common superscript within the same row were similar (*p* ≥ 0.05).

GPx, glutathione peroxidase; SOD, superoxide dismutase; TAC, total antioxidant capacity; MDA, malondialdehyde.

#### 3.3.5 Effects of thyroxine on apoptosis-like changes in post-thawed sperm

According to the data in Table 6, the group treated with T0.75 had the highest (*p* < 0.05) levels of viable sperm cells compared to the other groups. Conversely, the control group had the lowest (*p* < 0.05) levels of early apoptotic sperm cells compared to the other groups. Regarding late apoptotic sperm cells, all groups treated with thyroxine displayed significantly lower (*p* < 0.05) levels than the control group. Furthermore, all groups treated with thyroxine decreased (*p* < 0.05) the amount of necrotic sperm cells compared to the control group, with the T0.75 group showing the lowest value.

**Table 6:** Effect of different thyroxine additives on apoptosis-like changes of post-thawed buffalo bull sperm (Annexin V/PI assay).

Parameters/groups	Control	T0.25	T0.5	T0.75	T1
Viable (A−/PI−)	48.50 ± 0.76^d^	51.62 ± 1.00^c^	56.50 ± 0.63^b^	66.37 ± 0.65^a^	57.62 ± 0.68^b^
Early Apoptotic (A+/PI−)	5.37 ± 0.87^d^	12.25 ± 1.18^b^	16.12 ± 1.13^a^	8.88 ± 0.52^c^	13.50 ± 0.85^ab^
Late Apoptotic (A+/PI+)	32.87 ± 0.64^a^	27.75 ± 0.65^b^	22.25 ± 0.53^cd^	20.87 ± 0.54^d^	23.12 ± 0.64^c^
Necrotic (A−/PI+)	13.25 ± 0.53^a^	8.37 ± 0.37^b^	5.12 ± 0.40^c^	3.87 ± 0.29^d^	5.75 ± 0.36^c^

Number of ejaculates = 60. The means bearing one common superscript within the same row were similar (*p* ≥ 0.05).

Annexin V/PI assay (Annexin V and propidium iodide (PI) labeling of cells).

#### 3.3.6 Effects of thyroxine on cryosurvival rate and fertility of post-thawed sperm

**Figure 2:**
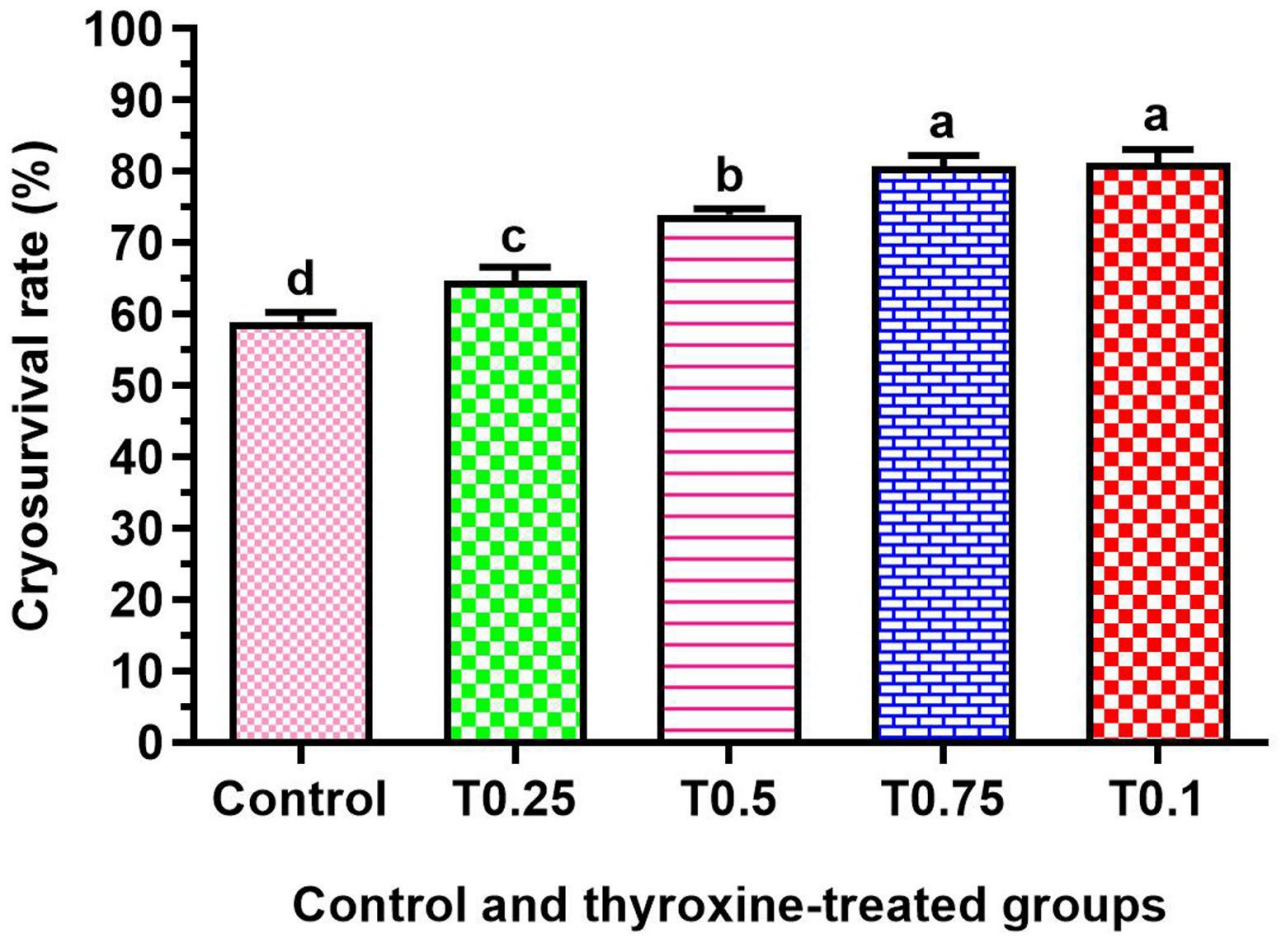
Effect of adding different thyroxin concentrations to the semen of buffalo bulls on cryosurvival rate. Number of ejaculates = 60. The data is presented as Mean ± SEM. Various superscripts (a, b, c) denote statistically significant variations within the columns where *p*-values are less than 0.05.

**Figure 3:**
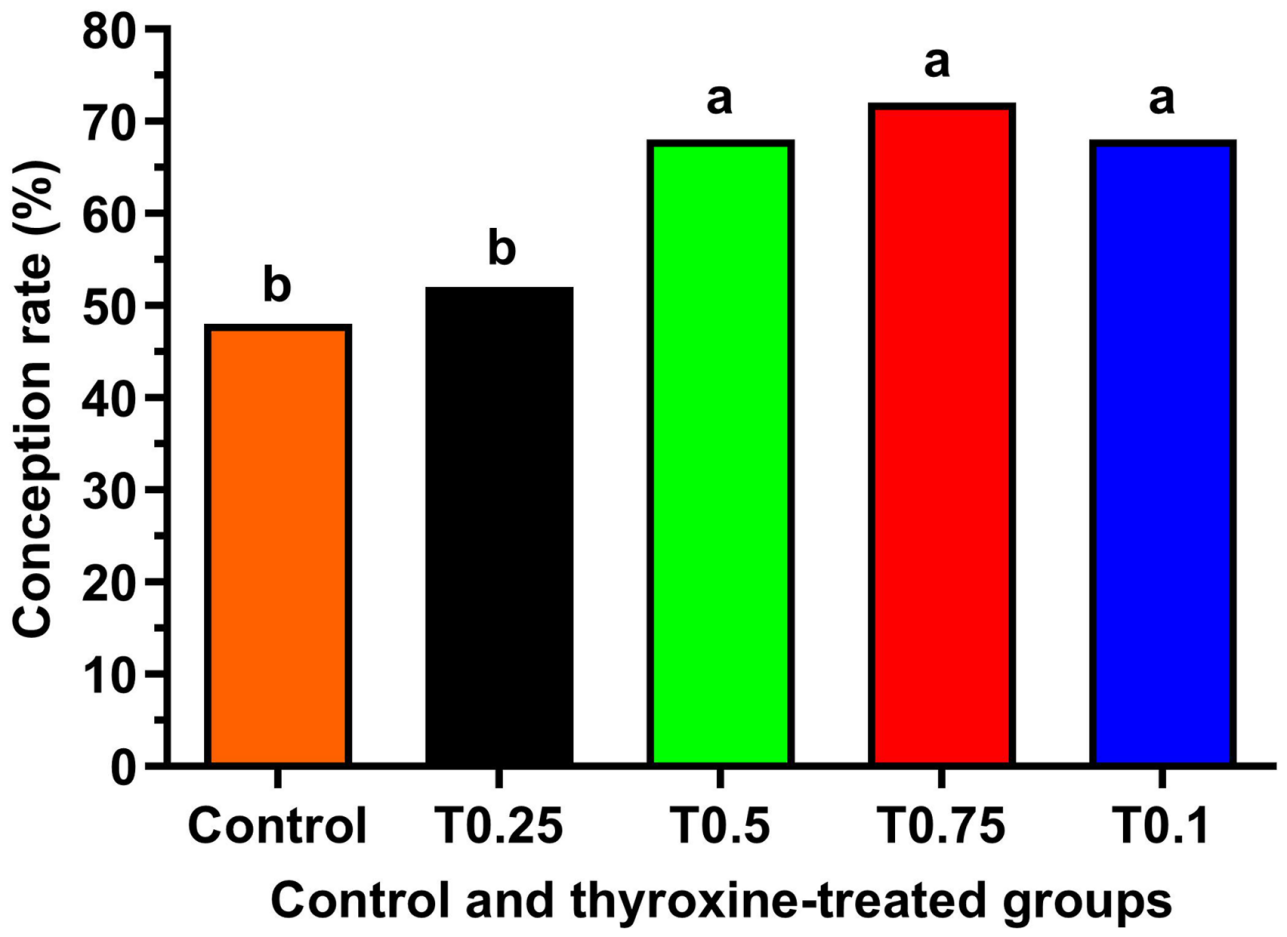
125 healthy cyclic buffaloes were divided into five groups, each containing 25 buffaloes. The conception rate of buffalo bull semen supplemented with different concentrations of thyroxine is shown. The data is presented as Mean ± SEM. Several superscripts (a, b) denote statistically significant variations within the columns where *p*-values are less than 0.05.

Figure 2 shows a significant increase in cryosurvival rate among all groups treated with thyroxine, with the T0.75 and T1 groups showing the most crucial improvement (*p* < 0.05). Additionally, the groups T0.5, T0.75, and T1 in Figure 3 demonstrated significantly higher conception rates (*p* < 0.05) compared to the control and T0.25 groups.

## 4 Discussion

This research examined how thyroxine affects the quality, function, and fertility of buffalo bull sperm in a lab setting at lower concentrations found in seminal fluid. This is the first time such a study has been conducted. We used doses lower than a physiological dose (1.39 ± 0.72 μg/dL) of T4. We tested lower doses at 0.1, 0.3, and 0.9 μg/dL, as well as higher doses at 2.7 and 8.1 μg/dL concentrations in this primary experiment.

The findings of this paper indicate that a lower physiological dose of thyroxine added to the semen extender improves total motility, progressive motility, and viability more than a higher physiological dose of thyroxine in equilibrated and frozen–thawed semen, consistent with the findings of La Vignera et al. (32) for human sperm. This could be attributed to the excessive consumption of substrates after exposure to higher concentrations of thyroxine, as noted by Xian et al. (33). Since the lower concentration proved more effective than the higher one, we conducted another main experiment to determine the optimal concentration of thyroxine that enhances sperm quality, functionality, and fertility.

These results revealed that in equilibrated semen, velocity parameters and viability percentage of the group treated with 0.75 μg/dL improved. In frozen–thawed sperm, improvements were observed in all aspects of motility, velocity, and viability percentage in the thyroxine-treated group compared to both the control group and the group treated with the lowest thyroxine concentration (0.25 μg/dL), consistent with the findings of Condorelli et al. (11) in human sperm, who reported increased motility (10%) in the thyroxine-treated group with a lower concentration of 0.9 pmol L^−1^ compared to the non-treated group. The potential explanation for this phenomenon could be the effects of thyroid hormones that do not involve genes, occurring when the hormones bind to receptors outside the cell nucleus in various parts of the sperm cell. This interaction stimulates cyclic adenosine monophosphate (cAMP) production and the release of calcium (Ca^2+^), ultimately leading to increased human sperm movement (32, 34). It has been observed that T4 can rapidly enhance the flagellar motion of sperm cells, inducing hyperactivity in human sperm cells (34).

Our results suggest that the T0.75 group has a positive impact on sperm performance in both equilibrated and frozen–thawed samples. This is evidenced by improvements in acrosome integrity, membrane integrity, MMP levels, and DNA integrity, which are consistent with the findings of Condorelli et al. (11). They reported that the increase in mitochondrial activity may be attributed to the enhancement of oxidative phosphorylation, which enhances sperm motility through a specific biochemical pathway until all available substrates are depleted. Conversely, an excessive dose of thyroxine can disrupt cytochrome C oxidase activity and reduce mitochondrial function, as demonstrated by Romano et al. (6). Additionally, thyroxine increases the quantity and functioning of mitochondria within cells by attaching to the DNA of the cells, thereby enhancing the basal metabolic rate (35).

The findings regarding the improvement of DNA quality align with research conducted by Meeker et al. (36) in human sperm, which suggests that T4 levels may have a protective effect on sperm DNA integrity. Several studies have indicated that high doses of thyroxine can cause DNA damage by increasing the production of reactive oxygen species (12) in human sperm. However, at levels close to normal physiological concentrations, thyroxine has been shown to decrease oxidative stress without affecting sperm DNA fragmentation and improve acrosome and plasma membrane integrity (11).

According to Saleh et al. (37), the cryosurvival rate better indicates human sperm quality after thawing than total or progressive motility. Adding 0.25, 0.5, 0.75, and 1 μg/dL of thyroxine to the extender before freezing significantly enhances the cryosurvival rate of buffalo sperm. This finding showcases the innovative approach of incorporating thyroxine before freezing buffalo bull sperm.

Some studies in buffalo bull sperm have shown that sperm cells exposed to cold shock, such as through cryopreservation, exhibit various structural changes when examined under a transmission electron microscope. These changes include increased plasma membrane fluidity, acrosome damage, and mitochondrial dysfunction (38, 39). Furthermore, these alterations in sperm morphology may trigger apoptosis and necrosis-like fluctuations during the cryo-storage and freeze–thaw processes (40) in Friesian bull’s sperm. This research revealed that thyroxine-treated groups showed improved viability and decreased late apoptotic and necrotic sperm cells. This improvement may be attributed to enhanced acrosome and sperm plasma membrane integrity and mitochondrial membrane potential. Conversely, mitochondrial dysfunction could lead to the initiation of buffalo sperm cell apoptosis and a decrease in fertilization capacity (41).

Our findings suggest enhancing the conception rate with buffalo bull semen can be achieved by supplementing it with various concentrations of thyroxine, particularly in the T0.5, T0.75, and T1 groups. We have shown that adding thyroxine to semen improves mitochondrial membrane potential, leading to increased ATP production by the mitochondria. This is essential for sperm viability and fertilization capability. The correlation between MMP and sperm motility is well established (42) in ram sperm, with both factors significantly determining human sperm fertilizing potential (43). The increase in MMP observed in this study aligns with improved mass activity and total progressive motility in frozen–thawed semen. Supplementation with thyroxine has been proven to enhance the integrity of sperm DNA, potentially leading to increased fertility in buffalo bulls, but the degradation of sperm DNA can negatively impact fertility (44).

## 5 Conclusion

Using a low concentration of thyroxine (lower than seminal plasma levels; 0.1, 0.3, and 0.9 μg/dL) in the extender for cryopreservation of buffalo bull sperm is highly beneficial. A concentration of 0.75 μg/dL of thyroxine has been shown to enhance sperm quality, functionality, and antioxidant capacity while reducing lipid peroxidation in the sperm. The group treated with 0.75 μg/dL of thyroxine exhibited the highest increase in sperm viability and the lowest number of dead sperm cells. Overall, all thyroxine treatments improved the survival rates of frozen–thawed sperm. It was also observed that the extender supplemented with thyroxine improved the fertility of frozen–thawed sperm in buffalo bulls.

## Data Availability

The original contributions presented in the study are included in the article/Supplementary material, further inquiries can be directed to the corresponding authors.

## Supplementary material

Supplementary TABLE S1 Extender preparation components.

Supplementary TABLE S2 Experimental design summary.

Supplementary IMAGE 1 Supplementary image for Eosin.

Supplementary IMAGE 2 Nigrosine stain method for detection of the sperm viability.

Supplementary IMAGE 3 Abnormalities.

SUPPLEMENTARY VIDEO 1 Sperm motility at T4 concentrations is 0.5.

SUPPLEMENTARY VIDEO 2 Sperm motility at T4 concentrations is 0.25.

SUPPLEMENTARY VIDEO 3 Sperm motility at T4 concentrations is 0.75.

Supplementary VIDEO 4 Sperm motility at T4 concentrations is 1.

Supplementary VIDEO 5 Sperm motility at T4 concentrations is control.
