# Supplementary material for: Optimizing thyroxine levels for enhanced buffalo sperm cryopreservation and fertility: a focus on quality, viability, and antioxidant protection
Source: Front Vet Sci. 2025 Apr 30;12:1584903. doi: 10.3389/fvets.2025.1584903 (PMC12075423; doi:10.3389/fvets.2025.1584903)
Supplement: Supplementary TABLE S1 — Extender preparation components. [file Data_Sheet_1.pdf]

Experimental Design Summary

Table 1 summarizes the extender preparation components and the two-phase experimental design used to evaluate the effect of different thyroxine concentrations on buffalo sperm quality and fertility.

Table S1. Extender Preparation Components

Component	Quantity (per 100 mL)
Tris	3.028 g
Citric acid	1.675 g
Fructose	1.25 g
Glycerol	6.0 mL
Egg yolk	20 mL
Penicillin	100 IU/mL
Streptomycin	100 µg/mL

Table S2. Experimental Design – Thyroxine Concentrations

Phase	Group	Thyroxine Concentration (µg/dL)
Primary	T0 (Control)	0
Primary	T0.1	0.1
Primary	T0.3	0.3
Primary	T0.9	0.9
Primary	T2.7	2.7
Primary	T8.1	8.1
Main	T0 (Control)	0
Main	T0.25	0.25
Main	T0.5	0.5
Main	T0.75	0.75
Main	T1	1
