# Supplementary figures and images for: Optimizing thyroxine levels for enhanced buffalo sperm cryopreservation and fertility: a focus on quality, viability, and antioxidant protection
Source: Front Vet Sci. 2025 Apr 30;12:1584903. doi: 10.3389/fvets.2025.1584903 (PMC12075423; doi:10.3389/fvets.2025.1584903)

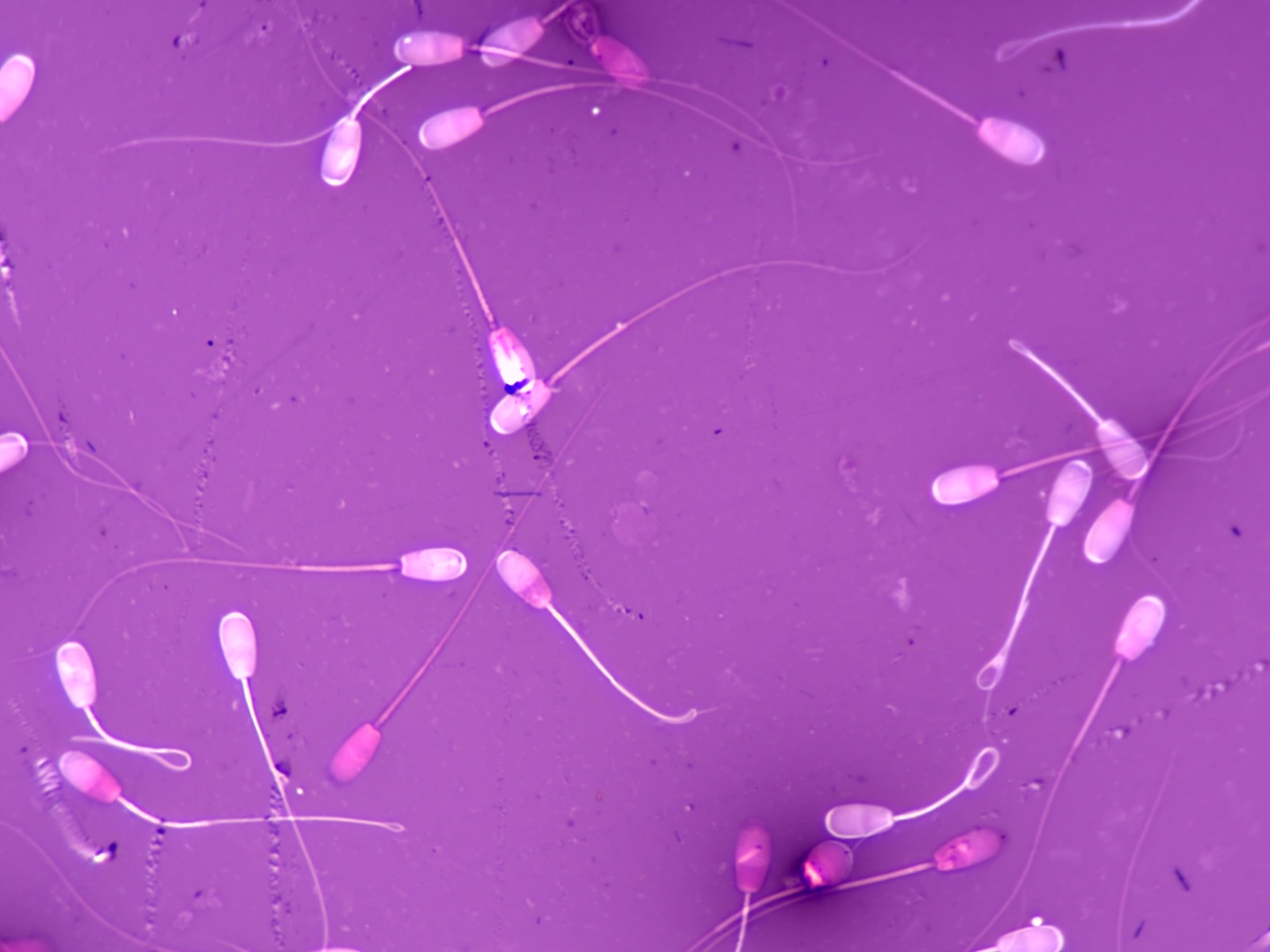

Supplement: Supplementary IMAGE 1 — Supplementary image for Eosin. [file Image_1.jpeg]

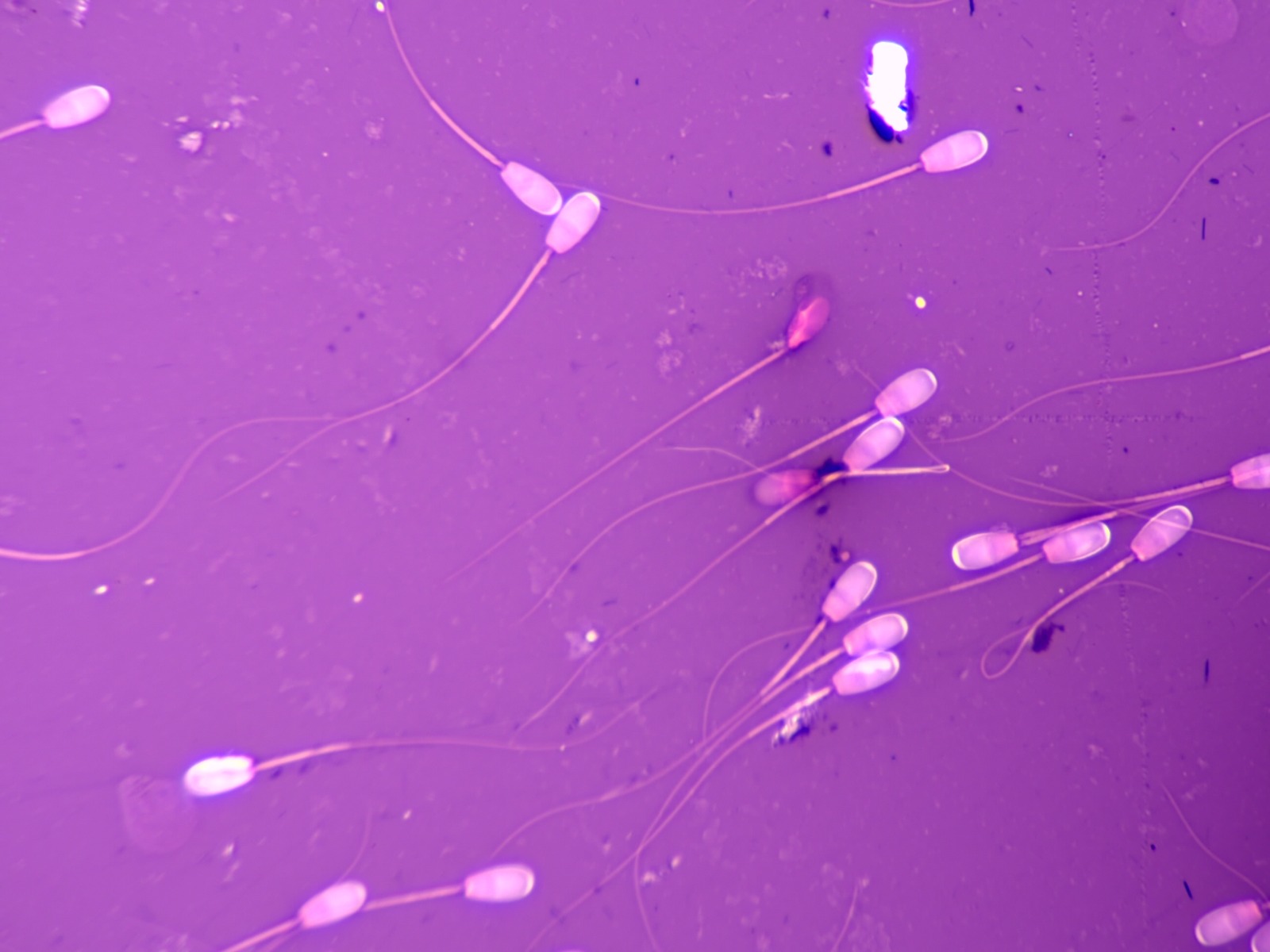

Supplement: Supplementary IMAGE 2 — Nigrosine stain method for detection of the sperm viability. [file Image_2.jpeg]

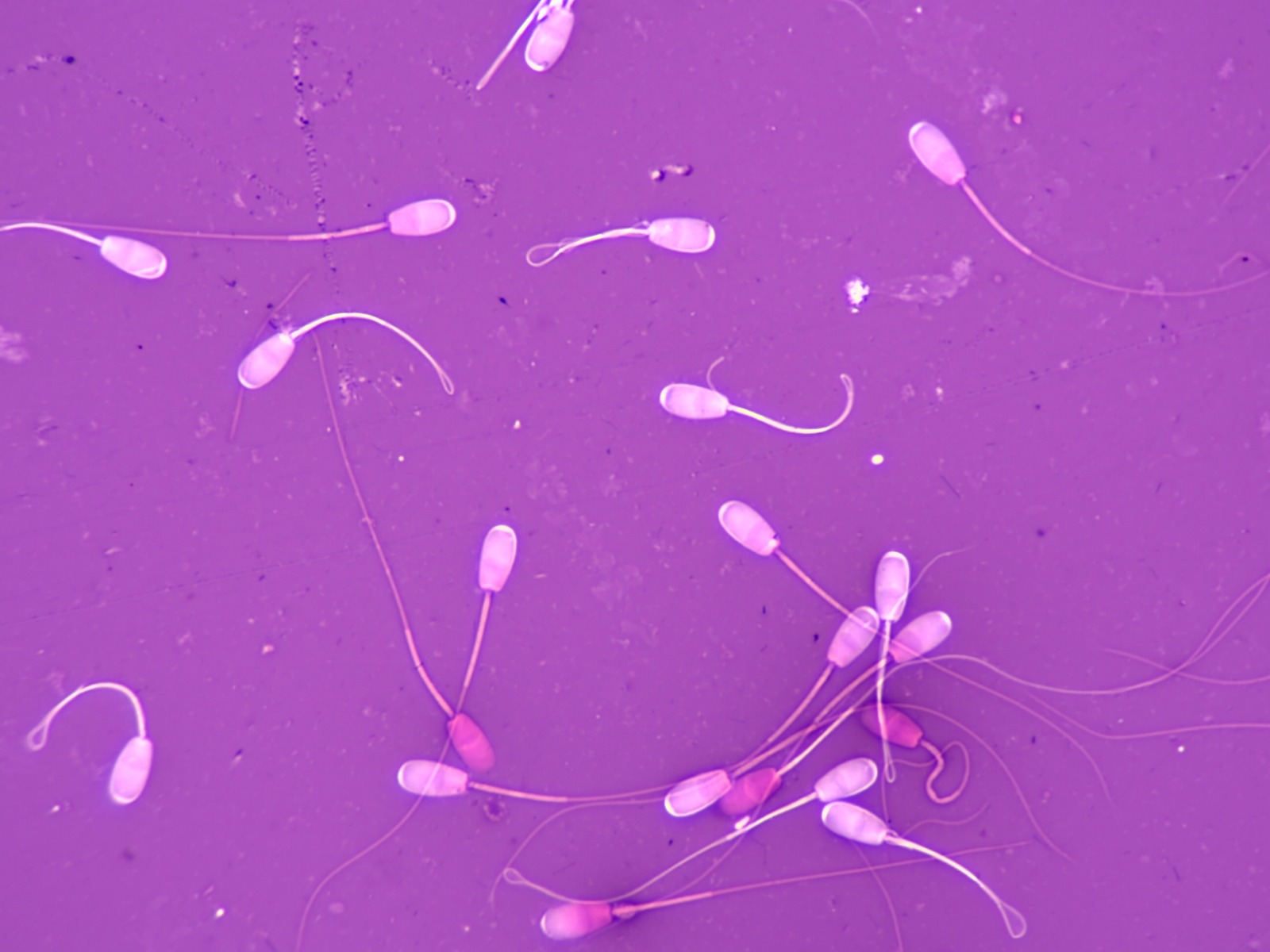

Supplement: Supplementary IMAGE 3 — Abnormalities. [file Image_3.jpeg]
